# A Split-Mouth Design Comparison for Lateral and Crestal Sinus Lift Techniques with Dental Implants Placements: Short Communication

**DOI:** 10.2174/1874210601711010603

**Published:** 2017-11-30

**Authors:** Saad Al-Almaie, Abdul Majeed Kavarodi, Ali Alorf, Saeed Alzahrani

**Affiliations:** 1 Medical Administration, King Fahd Military Medical Complex, Dhahran 31932, KSA; 2 Department of Dental Surgery, King Fahd Military Medical Complex, Dhahran 31932, KSA; 3 Department of Radiology, King Fahd Military Medical Complex, Dhahran 31932, KSA; 4 Department of Training and Education, King Fahd Military Medical Complex, Dhahran 31932, KSA

**Keywords:** Split-Mouth design, Sinus floor elevation, Lateral and crestal approaches, Visual analog scale, Implant placement, Peri-implant radiolucency

## Abstract

**Objective:**

The objective of this study is to compare and evaluate the effectiveness of implant placement and patient appraisal for two sinus lift techniques using both crestal and lateral techniques for bilateral sinus left in a split-mouth design.

**Introduction:**

All implants were successfully osseointegrated without any clinical complications or peri-implant radiolucency during the follow-up period of maximum 3 years.

**Methods:**

In terms of outcomes postoperative vertigo showed to be a major concern with the crestal approach, this approach is preferred over the lateral technique because of the reduced time required for the procedure and because it is less invasive.

**Results:**

Most patients preferred the crestal approach over the lateral approach due to the delay in implant placement.

## 1 INTRODUCTION

An implant-supported dental prosthesis can be a viable treatment option when there is sufficient quantity and quality of bone. The limitations of dental implant placement in the posterior maxilla, due to alveolar ridge resorption and excessive maxillary sinus pneumatization, can be overcome by maxillary sinus floor elevation [1].

Split-mouth designs first appeared in dental clinical trials in the late sixties. The main advantage of this study design is its efficiency in terms of sample size as the patients act as their own controls. Cited disadvantages relate to carry-across effects, contamination or spilling of the effects of one intervention to another, period effects if the interventions are delivered at different time periods, difficulty in finding similar comparison sites within patients and the requirement for more complex data analysis. Although some additional thought is required when utilizing a split-mouth design, the efficiency of this design is attractive, particularly in oral implantology studies where carry-across, period effects and dissimilarity between intervention sites does not pose a problem. Patients requiring both crestal (osteotome sinus floor elevation) and lateral antrostomy procedures on either side of the maxilla, as per current practice guidelines, constitute a very rare group. The quality and quantity of the maxillary residual ridge is the key factor in deciding between the two techniques in all patients [2]. Clinical and radiological assessments are utilized to evaluate the quality and quantity of the residual ridge. Both techniques have considerably variable procedural success rates, and patient’s appraisal of the techniques can also vary considerably. A split-mouth study, which is a self-controlled study design, is preferred here because it eliminates most of the sources of bias that occur in similar controlled studies [3-5].

The aim of this study was to compare and evaluate the effectiveness of implant placement and patient appraisal for two sinus lift techniques using both crestal and lateral techniques for bilateral sinus left in a split-mouth design by using the outcome variables for a visual analog scale (VAS) score for each question as patients’ symptoms, and peri-implant soft tissue conditions for both the lateral and crestal approaches.

## 2 MATERIALS AND METHODS

An approval for the study was granted by the Hospital Ethics Committee with a protocol number: kfmmc-dd18/05/2011(dated 18/May/2011, KFMMC, Dhahran, KSA.) The participants were informed about the aims and protocol of the study, and they provided proper consent. All bilateral sinus lift cases during the period from 2006 to 2012 were considered. Patients were selected according to the inclusion and exclusion criteria shown in Table **1**, [6-8]. From a group of sixty patients recruited in the same centre for another study [9]. Pre-operative records included: orthopantomography, intraoral photographs and Computed Tomography scans. The procedures are performed by the two surgeons in a hospital setup and the cases were allocated to both surgeons on a random fashion to avoid operational bias. Same bone graft material (Geistlich Bio-Oss®, Osteohealth Co. Shirley. NY 11967) and SLA, screw-type ITI implant (Institut Straumann Ch Company, Basel, Switzerland) were used in both cases. All patients were treated under Local Anaesthesia with same rotary instruments and were 4-0 vicryl sutures were used in all cases. No sutures were done in crestal approach sinus lift. Soft tissue parameters were obtained to compare the peri-implant soft tissue conditions and marginal bone levels between the two approaches. The patients’ appraisals of both techniques were assessed using a visual analog scale (VAS) based questionnaire. All patients were instructed to give their impressions and to evaluate, criticize and compare both surgical approaches for determining subjective maxillary sinus lift post-surgery expectations [10]. Patients were prescribed with both Ibuprofen 400 mg tablets four times orally in case of pain or Paracetamol 500mg tablets four times daily in case of pain.

## 3 RESULTS

The analysis of the data was performed using SPSS software, version 20.0. The results of the numeric responses are presented as the means ± standard deviations. The average VAS score for each question as patients’ symptoms for both sides were calculated and analyzed using Mann Whitney test. A timeline which was comparable for each surgical procedure was also recorded. At the annual clinical examination following functional loading, the survival of the implants and reconstructions were evaluated. Using two tailed T test, the averages of peri-implant soft tissue conditions for both the lateral and crestal approaches were evaluated clinically and radiographically.

## 4 DISCUSSION

A total of 20 sinuses lift procedures were performed in 10 patients. The mean follow-up period was 36 months after permanent prosthetic insertion. No complications with the surgical procedures were recorded, including infection of the maxillary sinus, loss of bone particles through the nose, wound dehiscence, and/or loss of the implants’ initial stability. All of the implants were stable at the latest follow-up, and all of the prostheses were functional. During the follow-up period, no pain or swelling were noted in any of the cases before or after prosthetic loading. All of the implants successfully fulfilled the Buser *et al*. criteria [11].

Apical elevation of the sinus floor for both approaches was observed. Conventional radiography proved bone/graft maturation around the implant apex in cases completed with the lateral approach, while for crestal (OSFE), no marked evidence was seen of bone formation between the lifted sinus membrane and the implant apex. There are no statistically significant differences in the marginal bone levels measured during radiological assessment between the lateral window and the OSFE implants. Pocket probing depth PPD is used to measure the distance between implant shoulder and mucosal margin DIM. Distance between implant shoulder and first visible bone implant contact DIB were analysed to compare the implant survival showed insignificant p values 0.0504, 0.7784, 0.1817 respectively.

**Fig. (1):**
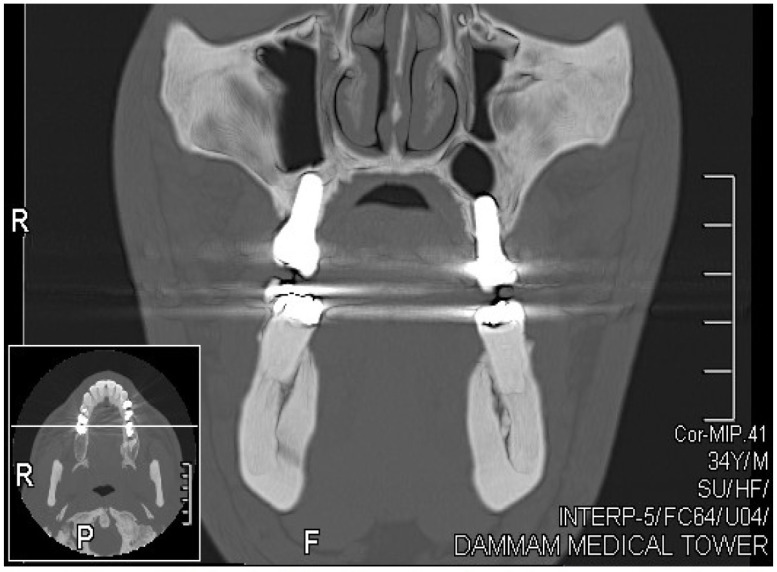
Coronal Computed Tomography showing bilateral bone formation, lateral approach in the right side and crestal approach in the left side.

CT scans were performed in all of the patients, confirming the amount of bone gained bilaterally by both techniques, as shown on right and left coronal CT scans (Fig. **1**). Radiological evidence of the presence of bone over the implant apex was proved by CT scans. On periapical radiographs, bone formation at the apex of the implant was not always confirmed in the OSFE cases, whereas with the lateral technique, sufficient bone could be observed above the implant apex. One of our ten cases had a membrane perforation during the lateral approach in this study. After three years of prosthetic loading, periapical radiographs showed a stable clinical situation in the area around the apices of the implants on both sides.

**Fig. (2):**
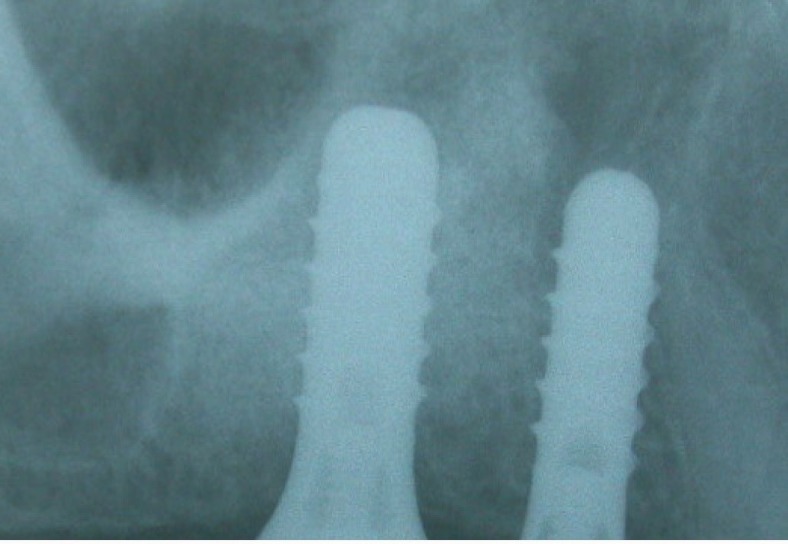
Three years post-operative periapical radiographs no reduced crestal bone resorptions were seen for lateral approach on right side.

**Fig. (3):**
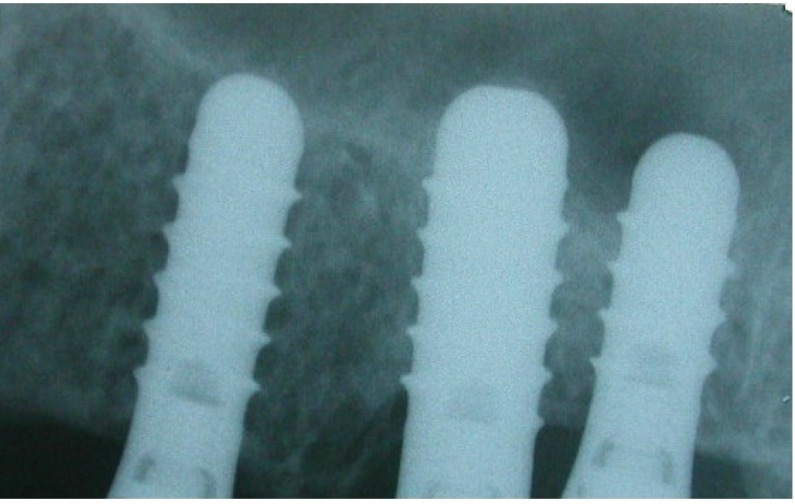
Three years post-operative periapical radiographs no reduced crestal bone resorptions were seen for crestal approach on left side.

No peri-implant radiolucency was noted in any of the cases (Figs. **2** and **3**). Because this was a split-mouth study, the comparison of both techniques in the same patient proved that the symptoms seen in the OSFE group alone may be specific to the technique. The crossover effect of learned memory in the split-mouth design was one of the limitations of this study [3, 12]. Checchi *et al*. [13] conducted a randomized clinical trial with a split-mouth design to compare the summers and cosci techniques in crestaly augmented sinuses with particulate cancellous human allografts.

In our study, all patients completed visual analog scale (VAS) based questionnaires listing the symptoms and complications in two stages, and the questionnaire was evaluated by a neutral analyst. As shown in Table **2**, mann-whitney test revealed that crestal approach was significantly associated with less severity pain, intraoral and extraoral swelling and bruising compared to lateral approach. In terms of vertigo the opposite was found. Crestal Approach was significantly associated with vertigo compared to lateral Approach. Interestingly, regarding the daily activity, the mean rank was less in crestal approach but there was no significant superiority compared to lateral approach. Similarly, but with opposite direction the lowest mean rank of lateral approach was less compared to crestal approach but this relation was not statistically significant.

Patients who experienced OSFE-associated symptoms during and after the OSFE did not experience any of these symptoms with the lateral approach. However, all of the patients recovered from these symptoms after 30 to 60 minutes. It was interesting to note that none of the patients were concerned about this transient discomfort, based on their responses. Although a delay in implant placement was the main reason for preferring OSFE over the lateral approach, the lateral approach was the choice of patients who feared the development of such symptoms and of patients who could not tolerate the discomfort of hammering. This finding also emphasizes the importance of disclosing all vestibular symptoms when presenting the OSFE procedure to the patient. The quality of the bone formed when replacing the bone graft with the lateral approach was different from the bone formed around the implant with the OSFE approach [14, 15]. However, such a comparison was beyond the scope of the study. Because no implants were lost, no significant differences were detected among the studied variables, and no reduced crestal bone resorption was seen on three years' postoperative radiographs; therefore, the procedures were considered to have attained implant success, per Buser *et al.* criteria [11]. This was a split-mouth study that attempted a precise, direct evaluation of both techniques for maxillary sinus lift [16]. This research was a part of the activity of research project was granted by the Ethics Committee of the Medical Association of the Military Hospital. The committee assembled after the completion of the study and also approved the study for publication.

## CONCLUSION

Maxillary sinus floor elevation with both the crestal and OSFE techniques could be evaluated precisely when both techniques were performed in the same patient. Although the small sample size for a split-mouth study was a limitation, the specific advantages, disadvantages and indications for each technique could be distinguished. The split-mouth design is a popular design in oral health research. The attractiveness of the design is to remove a lot of inter-individual variability from the estimates of the treatment effect.

## Figures and Tables

**Table 1:** The inclusion and exclusion criteria for Patients had been selected from a group of sixty patients recruited before in the same centre.

**Inclusion Criteria**	**Exclusion Criteria**
Patients required a bilateral sinus lift for implant treatment in the posterior maxilla one side for crestal approach and other side for lateral approach.	Patients who required a bilateral sinus, in which both the right and left sides were indicated for OSFE per current practice guidelines [6].
Patients were required to undergo a lateral sinus lift procedure if the RBS was a minimum of 2 mm and a maximum of 4 mm. Patients need to meet the current practice guidelines (minimum of 5mm RBS) to qualify for crestal approach^6^	Patients required a bilateral sinus, in which both the right and left sides were indicated for sinus lift by the lateral approach per current practice guidelines [7, 8].
At least 1 mm of bone was required on each side for implant stability.	Patients did not consent to participate in the study.
Patients were free from any systemic or local contraindications for dental implant placement.	Patient with systemic or local contraindications for dental implant placement.

**Table 2:** Mann-whitney test revealed that crestal approach was significantly associated with less severity pain, intraoral and extraoral swelling and bruising compared to lateral approach.

	Mean Rank		Sum of Ranks		*p* Value
	Crestal Approach	Lateral Approach	Crestal Approach	Lateral Approach	
Severity of Pain	5.65	15.35	56.50	153.50	0.000
Intraoral & Extraoral Swelling	5.50	15.50	55.00	155.00	0.000
Bruising	5.50	15.50	55.00	155.00	0.000
Vertigo	14.8	6.20	148.00	62.00	0.000
